# Poly(urethane-norbornene) Aerogels via Ring Opening Metathesis Polymerization of Dendritic Urethane-Norbornene Monomers: Structure-Property Relationships as a Function of an Aliphatic Versus an Aromatic Core and the Number of Peripheral Norbornene Moieties

**DOI:** 10.3390/molecules23051007

**Published:** 2018-04-25

**Authors:** Aspasia Kanellou, George C. Anyfantis, Despoina Chriti, Grigorios Raptopoulos, Marinos Pitsikalis, Patrina Paraskevopoulou

**Affiliations:** 1 Laboratory of Inorganic Chemistry, Department of Chemistry, National and Kapodistrian University of Athens, Panepistimiopolis Zografou, 15771 Athens, Greece; aspasiakan@hotmail.com (A.K.); chritides@chem.uoa.gr (D.C.); grigorisrap@chem.uoa.gr (G.R.); 2 Department of Materials Science, University of Patras, University Campus, 26504 Rio, Greece; gc.anyfantis@gmail.com; 3 Laboratory of Industrial Chemistry, Department of Chemistry, National and Kapodistrian University of Athens, Panepistimiopolis Zografou, 15771 Athens, Greece; pitsikalis@chem.uoa.gr

**Keywords:** aerogels, dendritic monomers, ring opening metathesis polymerization (ROMP), polymeric materials, ruthenium

## Abstract

We report the synthesis and characterization of synthetic polymer aerogels based on dendritic-type urethane-norbornene monomers. The core of those monomers is based either on an aromatic/rigid (TIPM/Desmodur RE), or an aliphatic/flexible (Desmodur N3300) triisocyanate. The terminal norbornene groups (three at the tip of each of the three branches) were polymerized via ROMP using the inexpensive 1st generation Grubbs catalyst. The polymerization/gelation conditions were optimized by varying the amount of the catalyst. The resulting wet-gels were dried either from pentane under ambient pressure at 50 °C, or from *t*-butanol via freeze-drying, or by using supercritical fluid (SCF) CO_2_. Monomers were characterized with high resolution mass spectrometry (HRMS), ^1^H- and solid-state ^13^C-NMR. Aerogels were characterized with ATR-FTIR and solid-state ^13^C-NMR. The porous network was probed with N_2_-sorption and SEM. The thermal stability of monomers and aerogels was studied with TGA, which also provides evidence for the number of norbornene groups that reacted via ROMP. At low densities (<0.1 g cm^−3^) all aerogels were highly porous (porosity > 90%), mostly macroporous materials; aerogels based on the aliphatic/flexible core were fragile, whereas aerogels containing the aromatic/rigid core were plastic, and at even lower densities (0.03 g cm^−3^) foamy. At higher densities (0.2–0.7 g cm^−3^) all materials were stiff, strong, and hard. At low monomer concentrations all aerogels consisted of discrete primary particles that formed spherical secondary aggregates. At higher monomer concentrations the structure consisted of fused particles with the size of the previous secondary aggregates, due to the low solubility of the developing polymer, which phase-separated and formed a primary particle network. Same-size fused aggregates were observed for both aliphatic and aromatic triisocyanate-derived aerogels, leading to the conclusion that it is not the aliphatic or aromatic core that determines phase separation, but rather the solubility of the polymeric backbone (polynorbornene) that is in both cases the same. The material properties were compared to those of analogous aerogels bearing only one norbornene moiety at the tip of each branch deriving from the same cores.

## 1. Introduction

Aerogels are ultralight porous solid materials derived from wet-gels by replacing their liquid component with a gas in such a way that the volume of the dry object is about equal to the volume of the parent wet-gel [1]. Owing to their low density and low thermal conductivity, the prime application of aerogels is in thermal insulation. Aerogels can be produced from many materials, but the most studied variety is based on silica (i.e., amorphous silicon dioxide). Silica aerogels, however, are fragile and difficult to handle materials. This has been corrected with a special class of composite materials that include organic polymers and are referred to as polymer-crosslinked aerogels [2]. Polymer-crosslinked aerogels redirected the way of thinking about robust aerogels: since the high mechanical strength of those materials came from the polymer coating over the inorganic (oxide) skeletal framework, pure polymers with the same nanostructure and interparticle connectivity should have similar mechanical properties. That hypothesis has been verified with several organic (polymeric) aerogels, including aerogels based on phenolic resins (e.g., resorcinol-formaldehyde) [3,4], polyureas [5], polyimides [6], polyamides (Kevlar^TM^-like) [7], polybenzoxazines [8], polyurethanes [9], and aerogels derived via Ring Opening Metathesis Polymerization (ROMP), including polynorbornene and polydicyclopentadiene [10,11,12]. ROMP-derived aerogels are hard and hydrophobic, as expected for pure hydrocarbons.

**Scheme 1:**
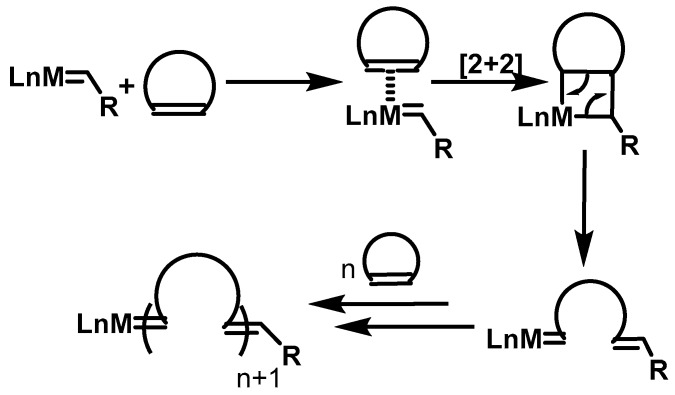
General mechanism of ROMP.

ROMP is one of the most prominent olefin metathesis reactions for cycloolefins and it is based on ring opening of monocyclic or polycyclic olefins followed by cross-coupling to form unsaturated polymeric chains (Scheme 1). Due to the abundance and variety of suitable monomers, ROMP has guided synthesis of a wide range of polymers and copolymers with diverse properties for numerous applications. The driving force of the ROMP reaction is ring strain release of cyclic alkenes, which therefore minimizes possible ring closing reactions [13,14]. ROMP is carried out with organometallic catalysts based on transition metals like W, Mo, Re, Ru, Ir, V, Ta or Ti [15]. Two of the main categories of catalysts are well-defined Schrock [16] and Grubbs [17] metal carbenes (tungsten, molybdenum and ruthenium complexes). The choice of catalyst depends on its ability to control molecular weights and their distributions, tolerance to high temperature and moisture, ability to polymerize monomers bearing functional groups, solubility in common organic solvents and ability to keep a polymerization “living” [18,19,20,21]. Also, depending on the catalyst (e.g., 1st or 2nd generation Grubbs catalyst, or mononuclear versus dinuclear tungsten compounds), the configuration of the double bonds along the polymer chain can vary (*cis*, *trans* or a mixture of both) [9,22,23,24,25,26,27,28,29,30], thereby allowing for further refinement of the properties to the resulting polymers/aerogels.

**Figure 1:**
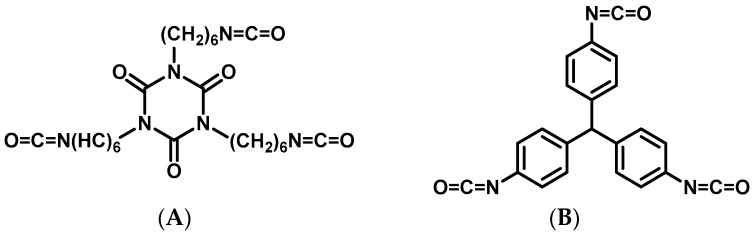
Structures of the triisocyanates contained in the commercial products referred to as Desmodur N3300 and Desmodur RE. In this publication the triisocyanate contained in Desmodur N3300 is referred to as N3300 (**A**) and the triisocyanate contained in Desmodur RE is referred to as TIPM (**B**).

The major requirement for the synthesis of organic polymer aerogels is phase separation of the polymer into tiny nanoparticles. That is ensured by crosslinking of the polymeric chains. In that regard, polynorbornene is a linear polymer and crosslinking is not favored. Better chances for crosslinking are found in polydicyclopentadiene via ring opening metathesis or radical coupling of the pendant cyclopentene rings. Yet, those processes are not controlled well. To alleviate those issues two or three norbornene moieties, able to develop polynorbornene chains independently, have been linked with hydrocarbon [31] or diimide bridges [32]. In a parallel venue, three norbornene moieties were linked, in a star-like fashion, to a well-defined trifunctional core with urethane bridges [33]. In order to understand the effect of increasing the number of norbornene moieties at the tips of branches emerging from trifunctional cores on the properties of the resulting aerogels, we expand along the lines of those previous studies with a dendritic monomer based on trifunctional core structures, linked again via urethane bridges to nine, rather than three, norbornene moieties, three at the tip of each branch. More specifically, the new dendritic urethane/norbornene monomers were synthesized, having an aromatic/rigid (triphenylmethane-4,4′,4′′-triisocyanate, TIPM, Desmodur RE) or an aliphatic/flexible (hexamethylene diisocyanate trimer, Desmodur N3300) triisocyanate core (Figure 1), and three NBE groups at the end of each of the three branches emanating from each core. The NBE moieties were polymerized via ROMP, using 1st generation Grubbs catalyst (GC-I). The resulting polymeric aerogels had a very high degree of crosslinking, high porosity and high thermal stability. The properties of those newly synthesized aerogels are compared with the properties of aerogels previously synthesized from similar trifunctional monomers bearing just one NBE group per branch [33].

## 2. Results and Discussion

### 2.1. Synthesis and Characterization of Dendritic Monomers aL-9-NBE and aR-9-NBE

**Figure 2:**
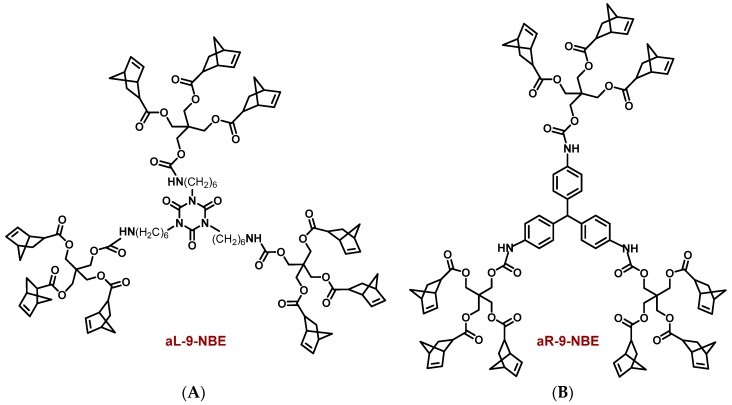
Structures of aL-9-NBE (**A**) and aR-9-NBE (**B**).

**Scheme 2:**
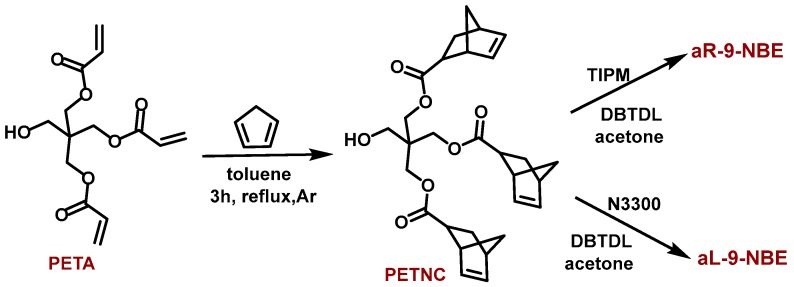
Synthesis of dendritic monomers.

Dendritic monomers aL-9-NBE and aR-9-NBE (Figure 2) were prepared via a two-step process (Scheme 2), which includes: (a) the synthesis of pentaerythritol-norbornene-carboxylate (PETNC) from pentaerythritol triacrylate (PETA) and cyclopentadiene (CPD) via a Diels-Alder reaction and (b) the synthesis of the dendritic monomers aL-9-NBE and aR-9-NBE from PETNC and an aliphatic (N3300) or an aromatic (TIPM) triisocyanate (Figure 1) via urethane formation using an organometallic tin catalyst (dibutyltin dilaurate, DBTDL). By design, those monomers include either an aliphatic/flexible or an aromatic/rigid core, and both bear nine terminal functional NBE groups (three NBE groups for each of the three branches). As described in the Introduction, similar star-shaped monomers featuring the same aliphatic or aromatic core, but with only three peripheral NBE groups (one NBE group at the tip of each of the three branches) have been reported recently [33].

**Figure 3:**
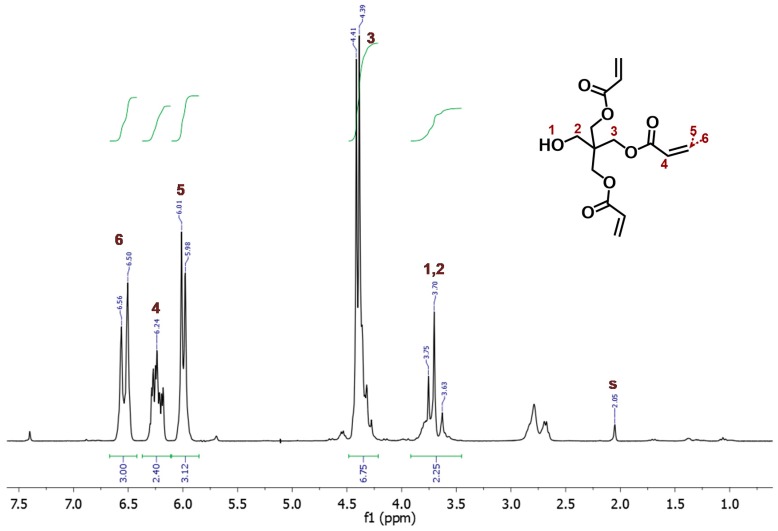
^1^H-NMR spectrum of SR444D in acetone-d_6_. The major component of this commercial product is pentaerythritol triacrylate (PETA), the peaks of which have been marked and integrated.

**Figure 4:**
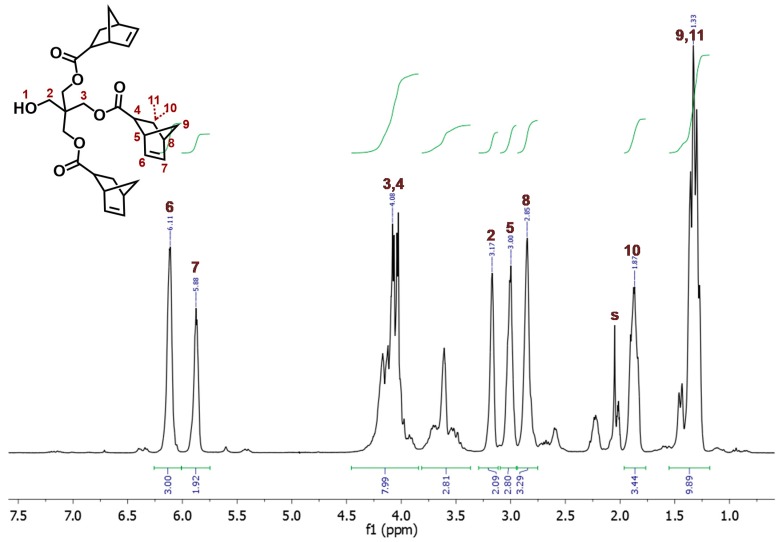
^1^H-NMR spectrum of pentaerythritol-norbornene-carboxylate (PETNC) in acetone-d_6_.

Figure 3 and Figure 4 show the ^1^H-NMR spectra of SR444D and PETNC, respectively. In the spectrum of SR444D the major peaks could be assigned to PETA, which is the major component of the commercial product SR444D, according to the literature [34]. The multiplets at 6.01, 6.24 and 6.50 ppm were assigned to the protons of the C=C double bond. Those resonances were not visible in the spectrum of PETNC and were replaced by two new singlets at 5.88 and 6.11 ppm, corresponding to the protons of the NBE C=C double bond, thus confirming the presence of NBE terminal groups. The other peaks of PETA (at 3.63, 3.70 and 4.39 ppm), attributed to aliphatic protons, remained practically unaffected in the spectrum of PETNC (observed at 3.61, 3.17 and 4.08 ppm, respectively). The additional peaks of the PETNC spectrum (at 1.33, 1.87, 2.85, 3.00 and 4.03 ppm) were assigned to the aliphatic protons of NBE.

**Figure 5:**
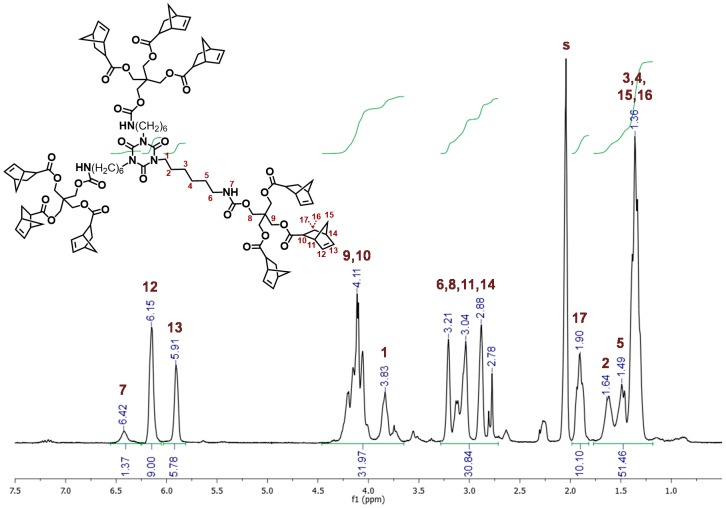
^1^H-NMR spectrum of aL-9-NBE in acetone-d_6_.

**Figure 6:**
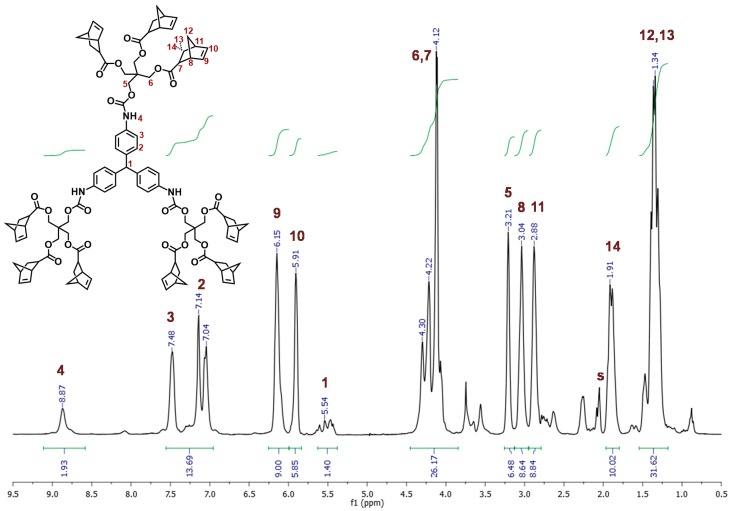
^1^H-NMR spectrum of aR-9-NBE in acetone-d_6_.

Monomers aL-9-NBE and aR-9-NBE were characterized with ^1^H- and ^13^C-CPMAS NMR, thermogravimetric analysis (TGA) and high-resolution mass spectrometry (HRMS). Figure 5 and Figure 6 show the ^1^H-NMR spectra of aL-9-NBE and aR-9-NBE. The peaks observed in those ^1^H-NMR spectra are in agreement with the expected structure of the monomers and with the peaks reported in the literature for similar monomers, bearing one NBE group per branch [33]. Peaks in the aliphatic region 1.3–1.7 ppm and at 3.21 and 3.83 ppm (Figure 5) were attributed to the aliphatic protons of Desmodur N3300, and the peaks at 5.54, 7.09 and 7.48 ppm (Figure 6) were assigned to the aromatic protons of TIPM, confirming the coupling of PETNC with the triisocyanates. Peaks at 5.91 and 6.15 ppm in the spectra of both monomers correspond to the NBE double bond.

**Figure 7:**
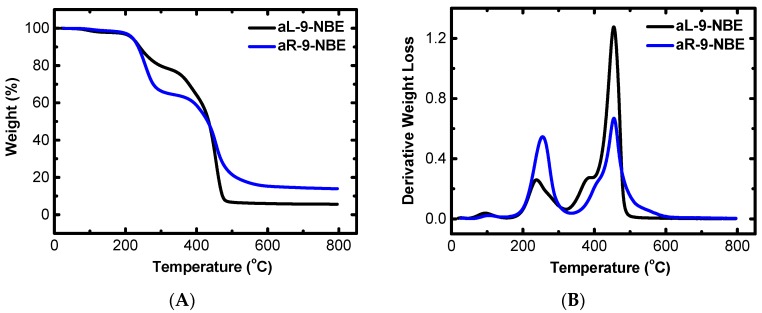
Weight loss with temperature (**A**) and derivative weight loss with temperature (**B**) for aL-9-NBE and aR-9-NBE monomers, as indicated.

Thermogravimetric analysis results are summarized in Figure 7 and Table 1. The DTG graphs showed two main decomposition events for the aliphatic monomer (aL-9-NBE) (238.8 °C and 454.2 °C, with a shoulder at 379.2 °C), indicating a complicated thermal decomposition mechanism. The aromatic monomer (aR-9-NBE) showed a bimodal decomposition pattern as well (254.9 °C and 456.1 °C, with a small shoulder at 409.6 °C). Based on literature data [35,36], the first decomposition step corresponds to a retro-Diels-Alder reaction of the NBE groups, leading to release of cyclopentadiene (CPD). That reaction has been documented to take place in one step at temperatures around 200 °C [31,32] and in some cases in two steps, the first one at around 200 °C, and the second one above 300 °C, and in some cases up to 410 °C [36]. From the data shown in Figure 7 it is observed that the retro-Diels-Alder reaction of aL-9-NBE takes place in two steps, one at 238.8 °C and the other one at 379.2 °C, while in the case of aR-9-NBE it takes place in one step, at 254.9 °C. This conclusion is further supported by calculating the number of mol of CPD that were released during that reaction. For aL-9-NBE the weight loss was 30% (by 380 °C), and for aR-9-NBE it was 35% (by 320 °C). Using the molecular weight of CPD (66.1 g mol^−1^) and the molecular weights of the two monomers (1994.36 g mol^−1^ and 1857.13 g mol^−1^, respectively), the number of mol of the CPD groups that were removed was calculated equal to 9.1 in the case of aL-9-NBE, and equal to 9.8 mol CPD/mol aR-9-NBE (Table 5). Those values are considered within error of the expected ones, and support the expected structures of the two monomers.

### 2.2. ROMP Synthesis of Aerogels

**Scheme 3:**
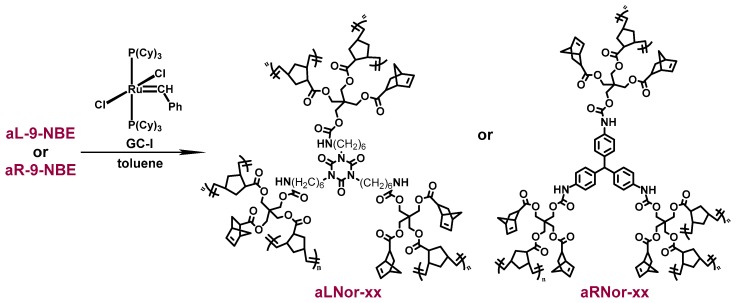
ROMP of aL- or aR-9-NBE monomers, catalyzed by 1st generation Grubbs catalyst (GC-I).

Aerogels were synthesized from the two dendritic monomers of Section 2.1 above via ROMP (Scheme 3). All gels were aged for 24 h. The polymerization of the monomers was quantitative as no free monomer could be detected by ^1^H-NMR in the washings of the resulting wet-gels with acetone. Three different parameters were varied: (a) the concentration of GC-I; (b) the drying method; and, (c) the monomer concentration. The resulting materials are labelled as aLNor-xx or ArNor-xx, where aL- and aR- refer to the aliphatic and the aromatic core, respectively, and -xx indicates the % *w/w* monomer concentration in the sol. The polymerization results for each of the two monomers and the characterization of the products are described below.

Different amounts of catalyst (GC-I) were tried for a constant concentration of the monomer (12% *w/w*), as shown in Table 2. Reducing the amount of catalyst increased the reaction time and gave unstable gels. Specifically, for [GC-I] = 0.12 mM (entry 4) no gelation was observed, while for [GC-I] = 0.6 mM (entry 3) the gels broke to pieces upon removal from the mold. For the highest GC-I concentration (2.4 mM, entry 1) the reaction time was very short, and the resulting gels were very rigid. An intermediate concentration of GC-I (1.2 mM; entry 2) did not compromise rigidity, while it allowed sufficient time until gelation for other manipulations (pouring to molds etc.). Therefore, that catalyst concentration was chosen for all subsequent experiments.

The effect of the drying method on the properties of the dry-gels was studied with wet-gels of aLNor-12 prepared under identical conditions (monomer concentration at 12% *w/w*, and molar ratio of GC-I/monomer at 1/50). Three different drying methods were tested: (a) from supercritical fluid (SCF) CO_2_, (b) freeze-drying from *t*-butanol [37,38], and (c) from pentane at 50 °C under ambient pressure [39]. The results are presented in Table 3. Drying from SCF CO_2_ provided dry-gels with the lowest density and shrinkage. Freeze drying gave somewhat higher shrinkage and significantly higher densities than SCF CO_2_ drying (0.8 g cm^−3^ vs. 0.5 g cm^−3^). Drying from pentane yielded materials with densities above 1.0 g cm^−3^. Based on those data, all subsequent work focused on materials dried from SCF CO_2_.

**Figure 8:**
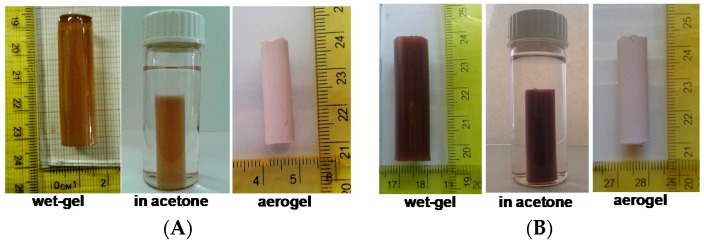
aLNor-xx (**A**) and aRNor-xx (**B**) before and after SCF CO_2_ drying.

Subsequently, ROMP of aL-9-NBE and aR-9-NBE was carried out at monomer concentrations of 1.5%, 3%, 6%, 12% and 15% *w/w*, a constant amount of GC-I as catalyst (10 mg) and toluene as solvent (10 mL). The formulations and gelation times of those reactions are presented in Table 4. From Table 4 it is obvious that the apparent gelation time for the aliphatic monomer was affected by the monomer concentration (especially at low monomer concentrations). On the other hand, for the aromatic monomer there was no immediate correlation between the apparent gelation time and the % *w/w* monomer concentration (Table 4). Nevertheless, by comparing apparent gelation times in Table 4, it is noted that the aromatic monomer gelled faster than the aliphatic one. Wet-gels were processed into aerogels (Figure 8), which were insoluble in common solvents and were studied with ^13^C CPMAS NMR spectroscopy, TGA, ATR-FTIR spectroscopy, SEM, N_2_-sorption porosimetry and helium pycnometry.

### 2.3. Characterization of Aliphatic and Aromatic Aerogels

**Figure 9:**
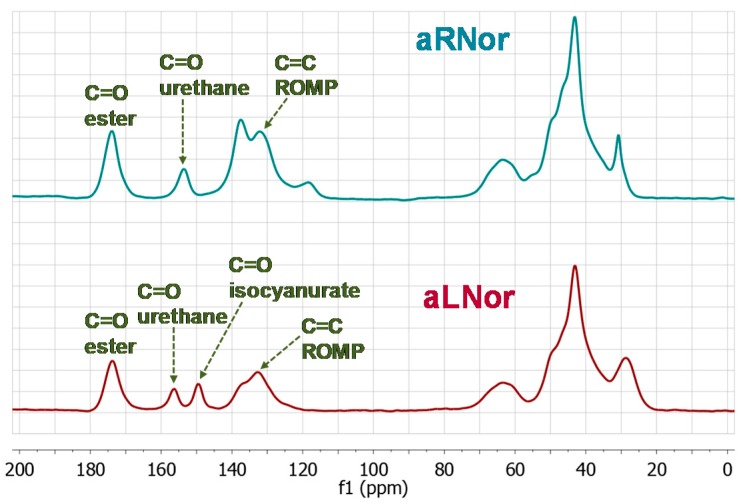
^13^C-CPMAS NMR spectra of aliphatic (aLNor) and aromatic (aRNor) aerogels.

Chemical characterization and identification of aerogels was done using ATR-FTIR and ^13^C- CPMAS NMR. Figure S1 shows the ATR-FTIR spectra of the aliphatic (aLNor) and the aromatic (aRNor) aerogels and the assignment of the main peaks. Characteristic peaks in the spectrum of aromatic aerogels (aRNor) are the stretching vibration of the urethane C–N bond at 1210 cm^−1^ and of the N–H bond at 3336 cm^−1^. The N–H bond shows a bending vibration coupled with the stretching vibration of the O–N bond at 1514 cm^−1^. The stretching vibration of aromatic C–C appears at 1596 cm^−1^ and that of the urethane carbonyl at 1729 cm^−1^. Similarly, for polymers obtained from the aliphatic monomer (aLNor), almost the same peaks are observed, with small differences. The stretching vibration of the isocyanurate carbonyl appears at 1689 cm^−1^, while the stretching vibration of the urethane C–N bond was shifted to 1233 cm^−1^. The stretching vibration of N–H with or/and without hydrogen bonding appeared at 3384 cm^−1^. Figure 9 shows the ^13^C-CPMAS NMR spectra of the aliphatic (aLNor) and the aromatic (aRNor) aerogels. In the ^13^C-CPMAS NMR spectra, the peak of the urethane carbonyl appeared at 154 ppm for aromatic samples and at 156 ppm for aliphatic samples. The ROMP-derived C=C double bond appeared at 133 ppm. ATR-FTIR and ^13^C-CPMAS NMR spectra are in agreement with the expected polymer structures and with the literature [33].

**Figure 10:**
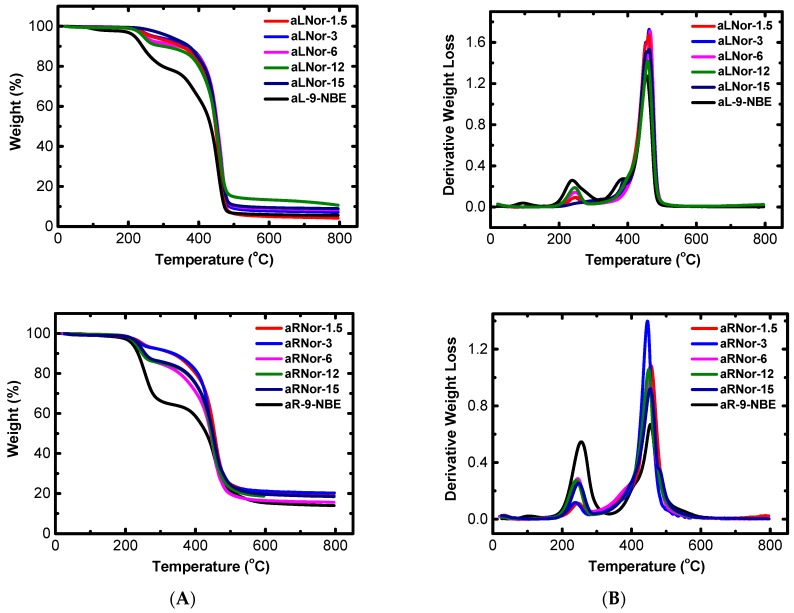
Weight loss with temperature (**A**) and derivative weight loss with temperature (**B**) for aLNor-xx and aRNor-xx aerogels, as indicated.

Through thermal characterization, it was observed that the thermal decomposition of all aerogels started after 150 °C (Figure 10) and was similar to the decomposition of the monomers (Figure 7), showing two main decomposition peaks. DTG peaks at 240–245 °C indicated a retro-Diels-Alder reaction, as in the respective monomers. That means that a certain number of NBE groups were not polymerized via ROMP. In turn that was attributed to crowding. Interestingly, it seems that the retro-Diels-Alder reaction for the aliphatic aerogels (aLNor) occurred in one step, at 240 °C, with the only exception of aLNor-15, for which this reaction occurred at 380 °C. As expected, in all cases those peaks corresponding to the retro-Diels-Alder were smaller compared to the ones for the monomers, and also smaller when monomer concentrations were lower and reaction rates were slower. Quantification, i.e., mol of CPD released, which is equal to mol of unreacted NBE, are shown in Table 5. Out of the 9 NBE groups present in the monomers, 2–4 remained unreacted for the aromatic monomer (aR-9-NBE), and 2–3 (6 for the highest concentration, 15% *w/w*) remained unreacted for the aliphatic monomer (aL-9-NBE).

Table 6 includes bulk and skeletal densities, porosities, BET surface areas and linear shrinkages for all aliphatic (aLNor-xx) and aromatic (aRNor-xx) samples. Due to the rigidity of the aromatic monomer, aromatic (aRNor-xx) aerogels exhibited lower linear shrinkages than aliphatic (aLNor-xx) aerogels (12–18% vs. 27–35%) and therefore lower densities (0.032–0.195 g cm^−3^ vs. 0.070–0.676 g cm^−3^), while for both classes of materials bulk densities decreased with decreasing concentration. All materials had high porosities, with those of the most dilute samples being as high as 96% (aLNor-1.5) or 98% (aRNor-1.5). Interestingly, aerogels based on the aliphatic/flexible core were fragile, whereas aerogels containing the aromatic/rigid core were plastic, and at low densities (0.03 g cm^−3^) foamy. At higher densities (0.2–0.7 g cm^−3^) all materials were stiff, strong, and hard.

**Figure 11:**
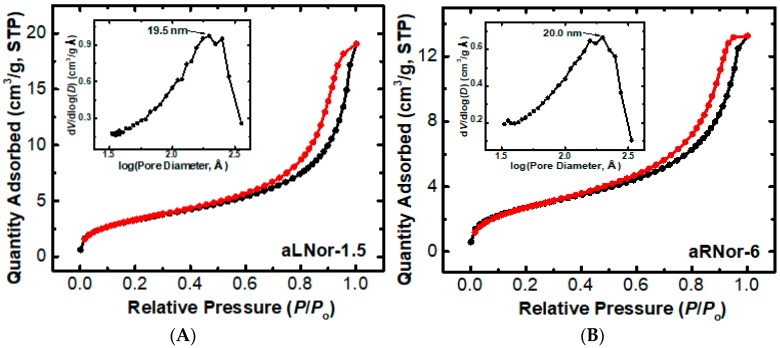
Representative N_2_-sorption diagrams for aliphatic (aLNor-1.5) (**A**); and aromatic (aRNor-6) (**B**). Insert shows the BJH distribution.

**Figure 12:**
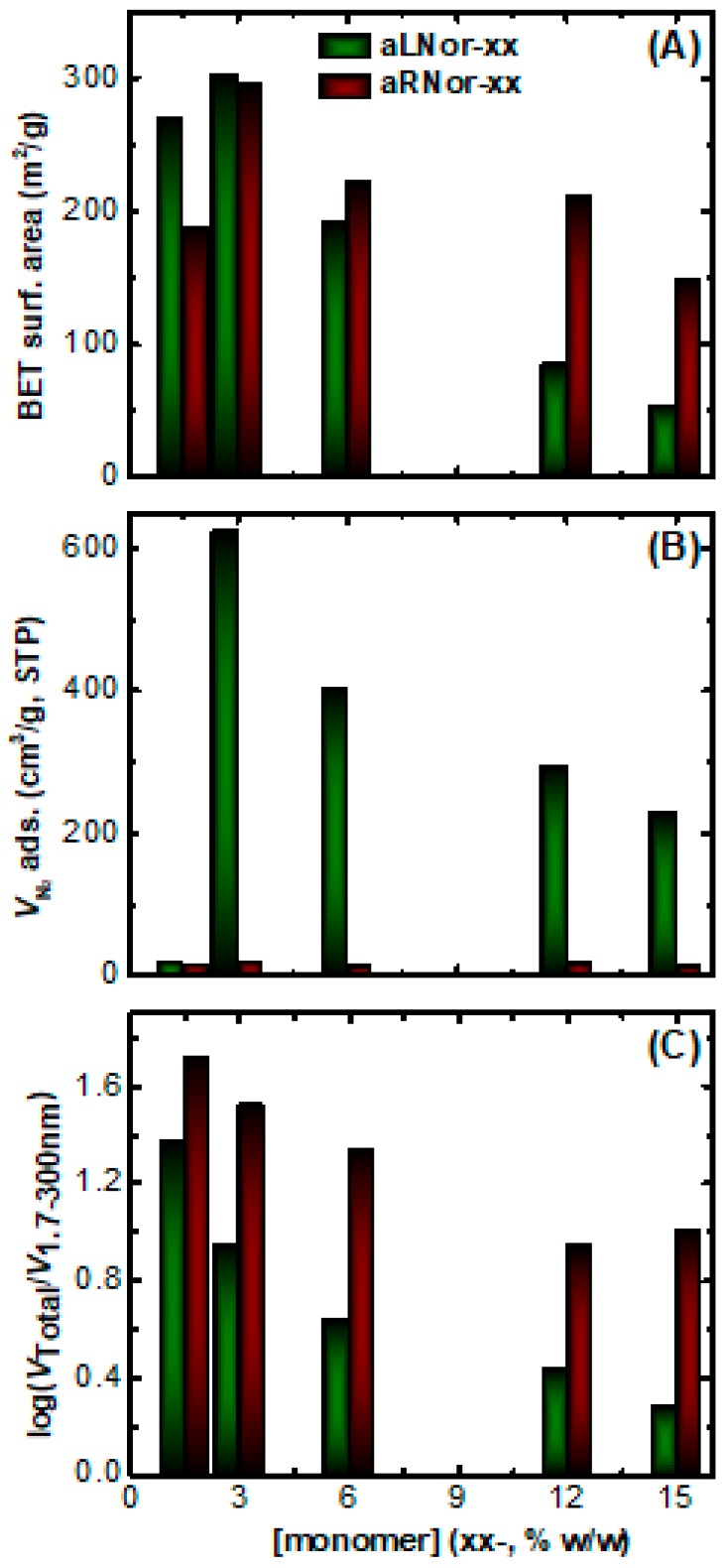
Comparative diagrams for data from N_2_-sorption porosimetry: BET surface area (**A**), total volume of N_2_ adsorbed (**B**), and *V*_Total_/*V*_1.7–300 nm_ ratio (**C**).

The porous network of the aerogels was examined with N_2_-sorption porosimetry. As shown in Figure 11, all N_2_-sorption isotherms for both the aliphatic (aLNor-xx) and aromatic (aRNor-xx) aerogels increased rapidly above *P/P*_0_ = 0.8 and exhibited narrow hysteresis loops, indicating mainly macroporous materials. Indeed, as shown in Table 6, *V*_Total_ >> *V*_1.7–300nm_, however as the monomer concentration in the sol increased the *V*_Total_/*V*_1.7–300nm_ ratio decreased (Figure 12), as expected for denser materials. Reflecting the lower shrinkage and lower density, thereby the higher rigidity, the total volume of N_2_ adsorbed was much higher for aRNor-xx samples. For the most part, low-density materials had higher macroporosities, while with increasing density, pore size decreased. For pores falling in the range of 1.7–300 nm, BJH curves showed maxima at 19.5 nm and 20.0 nm (insets in Figure 11). Those pore size distributions were rather broad as expected for networks formed via stochastic particle aggregation processes. The BET surface areas of aRNor-xx samples were higher than those of the aLNor-xx samples, and did not vary a lot with density. The surface areas of the aLNor-xx samples decreased as the density increased. As it is discussed below, the different behavior of the two types of materials is attributed to the different solubilities of the developing polymers, and to a secondary polymer accumulation on a primary particle network.

**Figure 13:**
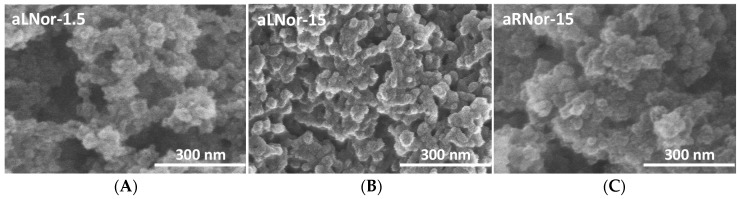
Representative SEM images of aLNor-1.5 (**A**), aLNor-15 (**B**), and aRNor-15 (**C**) (the two extreme sol concentration levels used in this study).

Microscopically, all aLNor-xx and aRNor-xx aerogels consist of random distributions of spherical nanoparticles. Representative SEM images are shown in Figure 13. The particle size varied depending on the monomer concentration in the sol. At low monomer concentrations (Figure 13A) the structure consisted of discrete tiny primary particles that formed spherical secondary aggregates. At higher monomer concentrations (Figure 13B) the finest particle definition was erased and the structure consisted of fused particles with the size of the previous secondary aggregates (at low monomer concentrations—Figure 13A). This structural evolution has been observed with several other polymeric aerogels before, and has been attributed to the low solubility of the developing polymer, which phase separates and forms a primary particle network, irrespective of the monomer concentration; if the latter is high, then a secondary polymer accumulation takes place on the primary network fusing particles together into frameworks like the one observed in Figure 13B [5,6,9]. Interestingly, same-size fused aggregates were also observed for aromatic triisocyanate-derived aRNor-15 (Figure 13C) leading to the conclusion that it is not the aliphatic or aromatic core that determines phase separation, but rather the solubility of the polymeric backbone (polynorbornene) that is in both cases (aLNor and aRNor) the same.

The evolution of the fine structure as inferred from SEM was cross-checked with direct particle size calculations from the gas sorption data of Table 6, using the relationship *r* = 3/(*ρ*_s_ × *σ*) (*r*: particle radius). Results are given in Table 7. Indeed, the particle radius of aLNor-15 jumped to a larger size (about 45 nm) consistent with secondary polymer accumulation within the secondary aggregates of a primary network that consisted of much smaller primary particles—on the order of the particle radii calculated for aLNor-1.5 to aLNor-12. Interestingly, despite the SEM similarity of aLNor-15 and aRNor-15, the secondary aggregates of the latter were still quite porous, and therefore the calculated particle radii were much smaller. That effect was considered to represent the direct contribution of the rigidity of the triisocyanate core on the solubility, and therefore the phase separation point of the developing polymer.

## 3. Materials and Methods

### 3.1. General Information

All procedures were carried out under an inert atmosphere, using Schlenk techniques on an inert gas/vacuum manifold or in a drybox (O_2_, H_2_O < 1 ppm). The inert gas used was Ar and it was passed through a BASF R-3-11 catalyst to remove traces of oxygen and moisture. Solvents and catalysts used were purchased from Alfa Aesar (Karlsruhe, Germany). Isocyanates (Desmodur N3300 and Desmodur RE, 27% TIPM solution in ethyl acetate) and pentaerythritol triacrylate (PETA, SR444D) were kindly provided by Covestro Deutschland GA (Leverkusen, Germany) and Sartomer Arkema Group (Rieux, France), respectively. Cyclopentadiene (CPD) was obtained by cracking of dicyclopentadiene (DCPD) dimer and was used within the next 24 h. All solvents were distilled under an inert atmosphere and were degassed via three freeze-pump-thaw cycles.

SCF drying was carried out in an autoclave (E3100, Quorum Technologies, East Sussex, UK). Samples for freeze-drying were kept in a Schlenk flask, were frozen in liquid nitrogen and then the flask was evacuated with a high-vacuum pump until all solvent was removed (at least 12 h).

^1^H-NMR spectra were obtained with a 300 Unity Plus spectrometer (Varian, Palo Alto, CA, USA) in deuterated acetone at room temperature. ^13^C-CPMAS NMR spectra were obtained with a 600 MHz Varian spectrometer operating at 150.80 MHz for ^13^C. For ^1^H-^13^C ramped Cross-Polarization Magic Angle Spinning (CPMAS) spectra, the spinning rate used was 5 kHz and the temperature for running the experiment was 25 °C. FTIR-ATR spectra were measured on an Equinox 55 (Bruker GmbH, Ettlingen, Germany) spectrometer, equipped with a single-reflection diamond ATR accessory DuraSamplIR II (SensIR Technologies, currently Smiths Detection, Edgewood, MD, USA). Scanning electron microscopy (SEM) was conducted with Au-coated samples, adhered on conductive double sided adhesive carbon tape, using a JSM-7500F field emission microscope (JEOL, Tokyo, Japan). The thermal stability of the polymers was studied by thermogravimetric analysis (TGA) employing a Q50 TGA model from TA instruments (TA Instruments-Waters LLC, New Castle, DE, USA). Samples were placed in platinum crucibles. An empty platinum crucible was used as a reference. Samples were heated from ambient temperatures to 800 °C in a 60 mL/min flow of N_2_ at a heating rate of 10 °C/min. N_2_-sorption measurements were made on a Micromeritics Tristar II 3020 surface area and porosity analyzer. Bulk densities (*ρ_b_*) of the samples were calculated from their weight and natural dimensions. Skeletal densities were determined by He pycnometry, using an AccuPyc II 1340 pycnometer (Micromeritics, Norcross, GA, USA).

Α QTOF mass spectrometer (Maxis Impact, Bruker Daltonics, Bremen, Germany) was used for the analysis and the identification of the monomers. The QTOF system was equipped with an electrospray ionization interface (ESI), operating in positive ionization mode, with the following operation parameters: capillary voltage 2500 V (PI); end plate offset, 500 V; nebulizer pressure 0.5 bar; drying gas 3 L min^−1^ and gas temperature 150 °C. The QTOF MS system operated in full scan acquisition mode and recorded spectra over the *m*/*z* range 500–2200, with a scan rate of 1 Hz. External calibration of the mass spectrometer was performed with the manufacturer’s solution (sodium formate clusters), reaching mass accuracy of 2–5 mDa. Infusion of the monomer solutions was performed under a constant flow of 180 μL min^−1^. Identification relied on the accurate mass measurement of the monomers. The identification criteria comprise a mass accuracy threshold of 5 mDa for the monoisotopic peak and a threshold (≤250 mSigma) for the isotopic pattern fit. mSigma-value is a measure for the conformity of fit between measured and theoretical isotopic pattern (the lower the mSigma value, the better the isotopic fit). The potential formation of adduct ions ([M + Na]^+^, [M + NH_4_]^+^) was taken into consideration.

### 3.2. Synthesis of Pentaerythritol-Norbornene-Carboxylate (PETNC)

Pentaerythritol triacrylate (PETA; 0.76 mL, 897 mg, 3 mmol), cyclopentadiene (CPD; 2.3 mL, 1808 mg, 27 mmol; PETA/CPD: 1/9 mol) and toluene (90 mL, 77.6 g) were added in a degassed Schlenk flask under Ar at r.t. The mixture was refluxed under stirring and Ar flow. After 3 h, the mixture was cooled at r.t. and the solvent was removed by distillation under vacuum, leaving PETNC. Yield: 97%. ^1^H-NMR spectrum (300 MHz, acetone-d_6_): δ (ppm) 6.11 (s, 3H), 5.88 (s, 2H), 4.17–3.98 (m, 8H), 3.17 (s, 2H), 3.00 (s, 3H), 2.85 (s, 3H), 1.87 (m, 3H), 1.36–1.30 (m, 10H).

### 3.3. Synthesis of aL-9-NBE and aR-9-NBE Monomers

The synthesis of aL-9-NBE and aR-9-NBE monomers was realized via the reaction of the triisocyanates Desmodur N3300 (1 g, 2 mmol) or Desmodur RE (27% solution of TIPM in ethyl acetate, 2.72 g, 2 mmol) with PETNC (3 g, 6 mmol), using DBTDL (10 µL, 10.7 mg, 0.017 mmol) as catalyst (DBTDL/triisocyanate: 1/120) in acetone (12 mL). The mixture was stirred at r.t. for 45 min under Ar. The solvent is then removed under vacuum. The crude product was redissolved in CH_2_Cl_2_ and hexane was added for precipitation, giving two phases: the upper phase was disposed, while the lower one (viscous oil) was dried under vacuum to provide the aliphatic or the aromatic monomer. *aL-9-NBE*: Yield: 60%. ^1^H-NMR (300 MHz, acetone-d_6_): δ (ppm) 6.42 (s, 3H), 6.15 (s, 9H), 5.91 (s, 9H), 3.90–4.4 (m, 27H), 3.83 (s, 6H), 3.21–2.78 (m, 30H), 1.90 (m, 9H), 1.64 (b, 6H), 1.49 (m, 6H), 1.43–1.20 (m, 39H). ^13^C-CPMAS NMR (100 MHz): δ (ppm) 173.8, 156.4, 149.4, 137.8, 132.8, 104.7, 88.5, 82.6, 74.5, 72.1, 64.8, 42.9, 28.7. HRMS: calcd for C_111_H_144_N_6_O_27_Na^+^: *m*/*z*_th_ = 2015.9971, *m*/*z*_exp_ = 2015.9972 (mass error = 0.1 mDa; Figure S2). *aR-9-NBE*: Yield: 50%. ^1^H-NMR (300 MHz, acetone-d_6_): δ (ppm) 8.87 (s, 3H), 7.48 (d, 6H),7.06 (m, 6H), 6.15 (m, 9H), 5.91 (s, 9H), 5.54 (m, 1H), 4.0–4.48 (m, 27H), 3.21 (s, 6H), 2.90–3.13 (s, 9H), 2.88 (s, 9H), 1.91 (m, 9H), 1.09–1.51 (m, 27H). ^13^C-CPMAS NMR (100 MHz): δ (ppm) 173.5, 153.0, 137.5, 132.1, 118.1, 104.6, 88.4, 82.8, 74.2, 71.9, 64.5, 55.0, 49.9, 45.9, 42.9, 35.0, 29.8. HRMS: calcd for C_109_H_121_N_3_O_24_Na^+^: *m*/*z*_th_ = 1879.8232, *m*/*z*_exp_ = 1879.8239 (mass error = 0.7 mDa; Figure S3).

### 3.4. Polymerization Reactions

Polymerizations were carried out at r.t. by adding a given amount of 1st generation Grubbs catalyst (GC-I) dissolved in a given amount of toluene and varying amount of monomer, depending on the desired concentration (% *w/w*) of the monomer in the solution. The mixture was left to react for 2–3 min under stirring, before being transferred into molds and left for 24 h. The resulting gels were solvent exchanged (4 × 8 h) with: (a) acetone before drying from supercritical fluid (SCF) CO_2_, (b) *t*-butanol before freeze-drying, or (c) pentane before drying at 50 °C under ambient pressure.

## 4. Conclusions

In summary, we prepared a series of synthetic polymer aerogels from two dendritic urethane-norbornene monomers. The core of those monomers consisted of either an aromatic/rigid (TIPM) or an aliphatic/flexible (N3300) triisocyanate. Those monomers featured norbornene (NBE) terminal groups (3 NBE groups at the tip each of the 3 branches of the monomer), which were polymerized via ROMP, using 1st generation Grubbs catalyst. Among the different drying procedures studied, i.e., from pentane at 50 °C, freeze-drying from *t*-butanol and SCF CO_2_ drying, the latter gave the best results (lower shrinkage and lower bulk density) to provide highly porous and mostly macroporous materials. All aerogels, irrespective of the aliphatic or aromatic core, consisted of discrete primary particles that formed spherical secondary aggregates (low monomer concentrations) and fused particles with the size of the previous secondary aggregates (high monomer concentrations), due to the low solubility of the developing polymer, which phase separates and forms a primary particle network.

Comparison of the ROMP reaction of the newly synthesized monomers (aL- and aR-9-NBE) with the ROMP of similar monomers (aL- and aR-3-NBE) from the literature, bearing one NBE group per branch, but the same aliphatic or aromatic core, showed that polymerization times (using GC-I catalyst) for the aromatic monomer (aR-9-NBE) were shorter than for the aliphatic one (aL-9-NBE), while the opposite trend was reported in the literature (using GC-II catalyst). It must be noted that our attempts to polymerize the aL- and aR-3-NBE monomers with GC-I, under the same experimental conditions reported in this work, were not successful, because of the insolubility of those monomers in toluene. Most importantly, the properties of the corresponding aerogels were very different. The newly synthesized aerogels have higher BET surface areas (60–300 m^2^ g^−1^) than their analogues based on 3-NBE capped star monomers (21–60 m^2^ g^−1^). High porosities and spherical particles appeared in both kinds of aerogels. Porosities were higher (68–98% vs. 44–89% in the 3-NBE capped analogues), but particles were smaller here (28 nm) vs. those in the 3-NBE-based aerogels (118 nm). All comparisons are made for aerogels from same or similar monomer concentration sols (10–20%).

Overall, the introduction of more peripheral NBE groups led to new materials with significantly improved properties, featuring higher crosslinking, larger porosities and higher surface areas than those reported before. To investigate further the effect of the number of peripheral NBE groups on the properties of the materials, the use of larger monomers, bearing five NBE moieties per branch, for the synthesis of aerogels is under study.

## Figures and Tables

**Table 1:** Thermal decomposition of aL-9-NBE and aR-9-NBE monomers.

Sample	Start (°C)	End (°C)	1st Peak (°C)	2nd Peak (°C)
aL-9-NBE	166.6	512.7	238.8	454.2
aR-9-NBE	156.3	628.9	254.9	456.1

**Table 2:** Polymerization reactions of aL-9-ΝΒΕ with different concentrations of GC-I catalyst ^1^.

Entry	GC-I (mg, [mmol])	[GC-I] (% *w*/*w*, [mM])	GC-I/Monomer (mol/mol)	Apparent Gelation Time ^2^ (min)
1	20 [0.024]	0.20 [2.4]	1/25	15
2	10 [0.012]	0.10 [1.2]	1/50	60
3	5 [0.006]	0.05 [0.6]	1/100	140
4	1 [0.0012]	0.01 [0.12]	1/500	-

^1^ aL-9-NBE: 1.2 g, 0.6 mmol, 12.2% *w/w*; solvent: toluene (10 mL); ^2^ Until the sol stopped flowing.

**Table 3:** Comparison of gels with different drying methods ^1^.

Sample	Linear Shrinkage ^5^ (%)	Bulk Density *ρ*_b_ (g cm^−3^)
aLNor-12-SCF ^2^	33 ± 5	0.5 ± 0.1
aLNor-12-FD ^3^	40 ± 1	0.8 ± 0.1
aLNor-12-PENT ^4^	53.45 ± 0.04	1.10 ± 0.02

^1^ aL-9-NBE: 12.2% *w*/*w*; GC-I/aL-9-NBE: 1/50 mol/mol; ^2^ Drying from SCF CO_2_; ^3^ Freeze-drying; ^4^ Drying from pentane at 50 °C; ^5^ Calculated according to the formula: (*y*_1_ + *y*_2_)/2, where *y*_1_ = 100 − [(*h*_fin_/*h*_st_) × 100] and *y*_2_ = 100 − [(*d*_fin_/*d*_st_) × 100; *h* = height, *d* = diameter.

**Table 4:** Polymerization reactions of dendritic monomers aL-9-ΝΒΕ and aR-9-NBE ^1^.

Sample	Monomer		Monomer	GC-I/Monomer	Apparent Gelation Time ^2^
	(g)	(mmol)	(% *w/w*)	(mol/mol)	(min)
aLNor-1.5	0.13	0.065	1.5	1/5	110
aLNor-3	0.30	0.150	3.4	1/12	65
aLNor-6	0.57	0.288	6.1	1/24	25
aLNor-12	1.15	0.576	11.2	1/48	20
aLNor-15	1.50	0.752	15	1/63	23
aRNor-1.5	0.12	0.065	1.5	1/5	10
aRNor-3	0.28	0.151	3.1	1/12	12
aRNor-6	0.54	0.291	5.4	1/24	35
aRNor-12	1.10	0.591	11.3	1/48	30
aRNor-15	1.42	0.765	14.3	1/63	15

^1^ GC-I: 10 mg, 0.012 mmol; [GC-I]: 0.11% *w/w*, 1.2 mM; solvent: toluene (10 mL). ^2^ Until the sol stopped flowing.

**Table 5:** Calculation of mol of CPD released via retro-Diels-Alder reaction during thermal decomposition of monomers aL-9-NBE and aR-9-NBE, and aerogels aLNor-xx and aRNor-xx.

-xx	aLNor-xx		aRNor-xx	
	CPD (mol)	CPD/aL-9-NBE (mol/mol)	CPD (mol)	CPD/aR-9-NBE (mol/mol)
monomer	0.454	9.1	0.526	9.8
1.5	0.100	2.0	0.109	2.0
3	0.085	1.7	0.118	2.1
6	0.123	2.4	0.222	4.1
12	0.148	3.0	0.216	4.0
15	0.318	6.4	0.211	3.9

**Table 6:** Properties of polymer aerogels aLNor-xx and aR-Nor-xx fabricated from the aliphatic (aL-9-NBE) and the aromatic (aR-9-NBE) monomer, respectively.

Sample	Linear Shrinkage ^1^ (%)	Bulk Density *ρ*_b_ (g cm^−3^)	Skeletal Density *ρ*_s_ (g cm^−3^)	Porosity ^2^ *Π* (% *v/v*)	BET Surf. Area *σ* (m^2^g^−1^)	*V*_Total_^3^ (*V*_1.7-300nm_) ^4^ (cm^3^ g^-1^)	Av. Pore Diameter ^5^ (nm)
aLNor-1.5	27 ± 3	0.070 ± 0.001	1.61 ± 0.08	96	270	13.66 (0.58)	88
aLNor-3	30 ± 1	0.125 ± 0.003	1.38 ± 0.03	91	302	7.28 (0.82)	13
aLNor-6	34 ± 8	0.24 ± 0.05	1.274 ± 0.009	81	191	3.38 (0.77)	13
aLNor-12	33 ± 5	0.5 ± 0.1	1.242 ± 0.005	60	85	1.19 (0.43)	24
aLNor-15	35 ± 1	0.676 ± 0.002	1.229 ± 0.005	45	54	0.66 (0.35)	24
aRNor-1.5	14 ± 1	0.032 ± 0.004	1.8 ± 0.1	98	188	30.69 (0.58)	123
aRNor-3	15 ± 2	0.045 ± 0.001	1.7 ± 0.1	97	294	21.63 (0.65)	88
aRNor-6	18 ± 2	0.10 ± 0.02	1.42 ± 0.02	93	221	9.30 (0.42)	78
aRNor-12	18 ± 1	0.17 ± 0.01	1.43 ± 0.04	88	211	5.18 (0.59)	11
aRNor-15	12 ± 1	0.195 ± 0.008	1.31 ± 0.01	85	148	4.36 (0.43)	116

^1^ Linear shrinkage calculated according to formula: (*y*_1_ + *y*_2_)/2, where *y*_1_ = 100 − [(*h*_fin_/*h*_st_) × 100] and *y*_2_ = 100 − [(*d*_fin_/*d*_st_) × 100; *h*: height, *d*: diameter; ^2^ Porosity calculated according to formula: (*ρ*_s_ − *ρ*_b_)/*ρ*_s_, where *ρ*_s_: skeletal density and *ρ*_b_: bulk density; ^3^ Volume calculated according to formula: 1/*ρ*_b_ − 1/*ρ*_s_. ^4^ Cumulative volume of pores between 1.7 and 300 nm from N_2_-sorption data and the BJH desorption method; ^5^ Calculated by the 4*V*/*σ* method; *V* was set equal to the maximum volume of N_2_ adsorbed along the isotherm as *P*/*P*_o_→1.0.

**Table 7:** Particle radii of aLNor-xx and aRNor-xx aerogels derived from N_2_-sorption and skeletal density data.

Sample-xx	Particle Radius *r* (nm)	
	aLNor-xx	aRNor-xx
1.5	6.9	8.9
3	7.2	6.0
6	12.3	9.6
12	28.4	9.9
15	45.2	15.5

## Supplementary Materials

The following are available online, Figure S1: ΑΤR-FTIR spectra of aliphatic (aLNor) and aromatic (aRNor) aerogels; Figure S2: Theoretical (blue) and experimental (black) mass spectra for the [M + Na]^+^ isotopes of aL-9-NBE. It is obvious that there is very good fit between the theoretical and experimental isotopic pattern (50 mSigma); Figure S3: Theoretical (blue) and experimental (black) mass spectra for the [M + Na]^+^ isotopes of aR-9-NBE. It is obvious that there is very good fit between the theoretical and experimental isotopic pattern (34 mSigma).
