# Supplementary material for: Poly(urethane-norbornene) Aerogels via Ring Opening Metathesis Polymerization of Dendritic Urethane-Norbornene Monomers: Structure-Property Relationships as a Function of an Aliphatic Versus an Aromatic Core and the Number of Peripheral Norbornene Moieties
Source: Molecules. 2018 Apr 25;23(5):1007. doi: 10.3390/molecules23051007 (PMC6102535; doi:10.3390/molecules23051007)
Supplement: Supplementary file 1 [file molecules-23-01007-s001.pdf]

molecules

Supporting Information

Poly(urethane norbornene) Aerogels *via* Ring Opening Metathesis Polymerization of Dendritic Urethane-norbornene Monomers: Structure-property Relationships as a Function of an Aliphatic Versus an Aromatic Core and the Number of Peripheral Norbornene Moieties

Aspasia Kanellou [1], George C. Anyfantis [2], Despoina Chriti [1], Grigorios Raptopoulos [1], Marinos Pitsikalis [3] and Patrina Paraskevopoulou [1,*]

[1] Laboratory of Inorganic Chemistry, Department of Chemistry, National and Kapodistrian University of Athens, Panepistimiopolis Zografou, Athens 15771, Greece; aspasiakan@hotmail.com (A.K.); chritides@chem.uoa.gr (D.C.); grigorisrap@chem.uoa.gr (G.R.); paraskevopoulou@chem.uoa.gr (P.P.)
[2] Department of Materials Science, University of Patras, University Campus, Rio 26504, Greece; gc.anyfantis@gmail.com (G.C.A.)
[3] Laboratory of Industrial Chemistry, Department of Chemistry, National and Kapodistrian University of Athens, Panepistimiopolis Zografou, Athens 15771, Greece; pitsikalis@chem.uoa.gr (M.P.)
* Correspondence: paraskevopoulou@chem.uoa.gr (P.P.); Tel.: +30-210-727-4381; Fax: +30-210-727-4782

Contents

		Page
**Figure S1**	ATR-FTIR spectra of aliphatic (aLNor) and aromatic (aRNor) aerogels.	S2
**Figure S2**	Theoretical (blue) and experimental (black) mass spectra for the $[M+Na]^+$ isotopes of aL-9-NBE. It is obvious that there is very good fit between the theoretical and experimental isotopic pattern (50 mSigma).	S2
**Figure S3**	Theoretical (blue) and experimental (black) mass spectra for the $[M+Na]^+$ isotopes of aR-9-NBE. It is obvious that there is very good fit between the theoretical and experimental isotopic pattern (34 mSigma).	S2

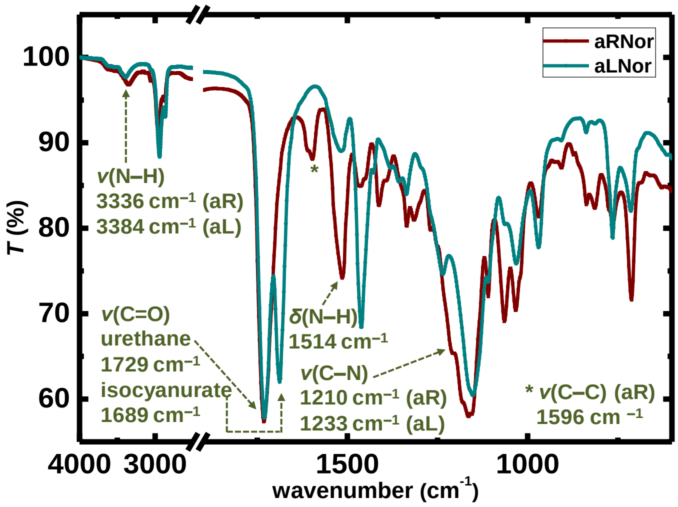

Figure S1: ATR-FTIR spectra of aliphatic (aLNor) and aromatic (aRNor) aerogels.

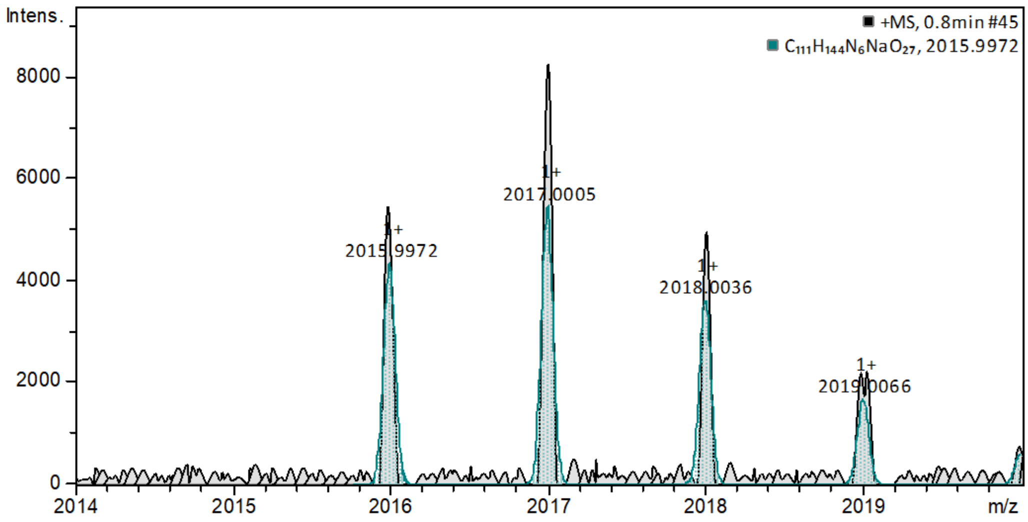

Figure S2. Theoretical (blue) and experimental (black) mass spectra for the $[M+Na]^+$ isotopes of aL-9-NBE. It is obvious that there is very good fit between the theoretical and experimental isotopic pattern (50 mSigma).

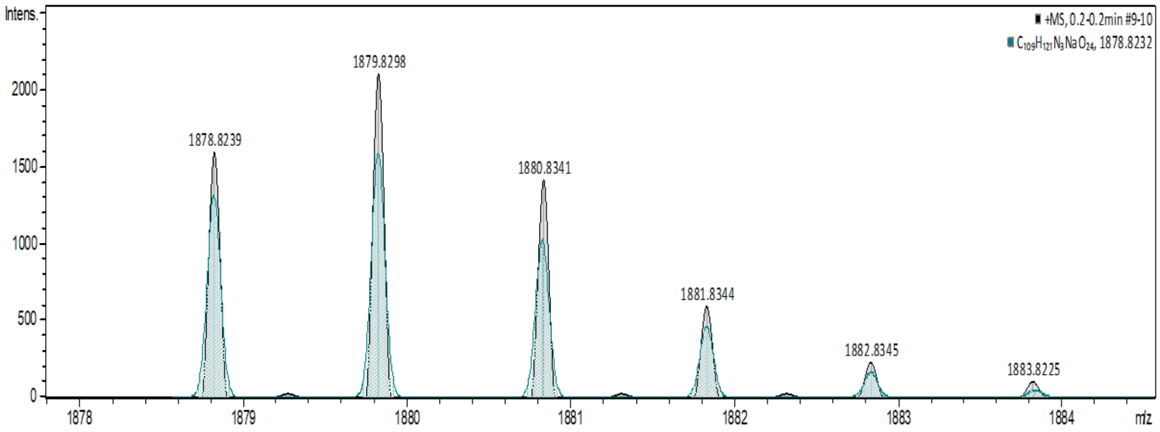

Figure S3. Theoretical (blue) and experimental (black) mass spectra for the $[M+Na]^+$ isotopes of aR-9-NBE. It is obvious that there is very good fit between the theoretical and experimental isotopic pattern (34 mSigma).
